# Association between abdominal hernia and the risk of subsequent dementia

**DOI:** 10.1002/brb3.1434

**Published:** 2019-10-06

**Authors:** Kuo‐Chuan Hung, Cheuk‐Kwan Sun, Jen‐Yin Chen, Hsiang‐Chi Wang, Chia‐Hung Kao

**Affiliations:** ^1^ Department of Anesthesiology Chi Mei Medical Center Tainan Taiwan; ^2^ Department of Emergency Medicine School of Medicine for International Students E‐Da Hospital I‐Shou University Kaohsiung Taiwan; ^3^ Department of Senior Citizen Service Management Chia Nan University of Pharmacy and Science Tainan Taiwan; ^4^ Management Office for Health Data China Medical University Hospital Taichung Taiwan; ^5^ College of Medicine China Medical University Taichung Taiwan; ^6^ Graduate Institute of Biomedical Sciences College of Medicine China Medical University Taichung Taiwan; ^7^ Department of Nuclear Medicine and PET Center Center of Augmented Intelligence in Healthcare China Medical University Hospital Taichung Taiwan; ^8^ Department of Bioinformatics and Medical Engineering Asia University Taichung Taiwan

**Keywords:** cohort study, dementia, hernia, matrix metalloproteinases

## Abstract

**Objective:**

Matrix metalloproteinases (MMPs) may play a role in the pathophysiology of neurodegenerative disease and hernia formation. This retrospective cohort study was designed to assess whether there is an association between hernia and the risk of dementia.

**Materials and Methods:**

Patients (≥45 years) with hernias were identified between 2000 and 2008 from a longitudinal claims data of one million beneficiaries from Taiwan's National Health Insurance program. A control group of patients with comparable distributions of sex, age, socioeconomic status, urbanization, and medical comorbidities without hernia were chosen for matching in a ratio of 1:1. Patients previously diagnosed with dementia were excluded. Follow‐up ended on December 31, 2013. Incidence rate of dementia was compared between patients with hernias and those without. Cox proportional hazards models were used to estimate hazards relative to those of the control group.

**Results:**

After matching, there were 4,784 hernia and 4,784 nonhernia patients. Hernia patients showed a higher incidence rate and hazard ratio of dementia than those in nonhernia group (8.82 vs. 7.19/1,000 person‐years; adjusted hazard ratio [aHR], 1.24; 95% CI, 1.07 to 1.45; *p* < .01). Advanced age (*p* < .0001), hypertension (*p* = .0139), head injury (*p* = .0003), and stroke (*p* = .041) were found to be risk factors for dementia, while patients with high socioeconomic status (*p* < .01) and history of coronary artery disease (*p* = .0292) were unlikely to develop dementia in our cohort study.

**Conclusion:**

Patients with hernias were associated with a higher incidence of dementia than those without. Our finding should be validated in further prospective studies with larger samples.

## Graphical abstract

Hernia patients had a higher incidence rate and hazard ratio of dementia than those in non‐hernia group. Advanced age, hypertension, head injury, and stroke were found to be risk factors for dementia. Patients with high socioeconomic status and history of coronary artery disease were unlikely to develop dementia in our cohort study.

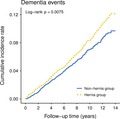

## 1 INTRODUCTION

Dementia, which is one of the most common neurodegenerative diseases, is characterized by its slow progressive impairment of memory together with at least one more cognitive domain (Caselli, 2003). Nonvascular dementia (e.g., Alzheimer's disease [AD]), which accounts for 80%–90% of cases, is the most common type followed by vascular dementia and Lewy body dementia (Morris, 2003). The prevalence of dementia tends to increase with aging, while a growing body of evidence supported a variety of disorders (e.g., diabetes, hypertension, ischemic heart disease, chronic obstructive pulmonary disease, heart failure, depression, dyslipidemia, head injury, and stroke) as risk factors (Kohler, Buntinx, Palmer, & Akker, 2015; Poblador‐Plou et al., 2014; Qiu, Xu, & Fratiglioni, 2010; Sahathevan, Brodtmann, & Donnan, 2012). It was estimated that the number of demented patients will double every 20 years, reaching 65.7 million by the year 2030 (Prince et al., 2013), thereby imposing substantial social and financial burdens on global health services.

Multiple neuropathologic processes involved in neurodegenerative and vascular diseases may contribute to the development of dementia. Previous studies have proposed an important role of matrix metalloproteinases (MMPs) in precipitating pathological changes in the brain and the blood–brain barrier (e.g., blood–brain barrier leakage, neuroinflammation, and neurodegeneration; Kim & Joh, 2012; Mroczko, Groblewska, & Barcikowska, 2013; Rempe, Hartz, & Bauer, 2016; Rosenberg, 2009; Weekman & Wilcock, 2016). For example, elevated levels of MMP‐9 as well as changes in the MMP/tissue inhibitors of MMPs (TIMP) balance in the plasma of AD patients were found (Lorenzl et al., 2003; Lorenzl, Buerger, Hampel, & Beal, 2008). Another study demonstrated that imbalances between TIMPs and MMPs are involved in blood–brain barrier breakdown and are implicated in the human immunodeficiency virus (HIV) type 1‐associated neurocognitive disorders (Xing et al., 2017).

The pathogenesis underlying abdominal wall hernia is multifactorial, and current evidence suggests that the formation of abdominal hernia may be associated with disturbances in extracellular matrix turnover and connective tissue metabolism (Antoniou et al., 2011; Antoniou, Antoniou, Granderath, & Simopoulos, 2009; Franz, 2008; Henriksen, Yadete, Sorensen, Ågren, & Jorgensen, 2011). For instance, MMPs (e.g., MMP‐1, MMP‐2, MMP‐9, MMP‐13) appear to be involved in inguinal hernia or recurrent inguinal hernia development (Antoniou et al., 2011, 2009). Considering that the pathogenesis for both hernia and dementia may be associated with MMPs, there may be a missing link between hernias and dementia. We hypothesize that adult patients with hernias may be at an increased risk for dementia. To test this hypothesis, we conducted a nationwide, population‐based cohort study to examine the relationship between middle‐aged (i.e., 45 years or older) patients with hernias and dementia.

## 2 METHODS

### 2.1 Data source

The National Health Insurance (NHI) program, which had a coverage rate of approximately 99% under mandatory enrollment, was initiated in 1995 for the purpose of providing comprehensive healthcare for residents and citizens in Taiwan. The present study adopted Longitudinal Health Insurance Database (LHID) for analysis, which is a subdatabase of the NHI program with one million individuals and their information of demographic characteristics, inpatient admissions, outpatient visits, and drug prescriptions. For all subjects in the database, the International Classification of Diseases, Ninth Revision, Clinical Modification (ICD‐9‐CM) was applied to identify their disease histories. The Research Ethics Committee of China Medical University and Hospital (CMUH104‐REC2‐115‐CR3) reviewed and approved the protocol of the present study.

### 2.2 Identification of study patients and control group

**Figure 1:**
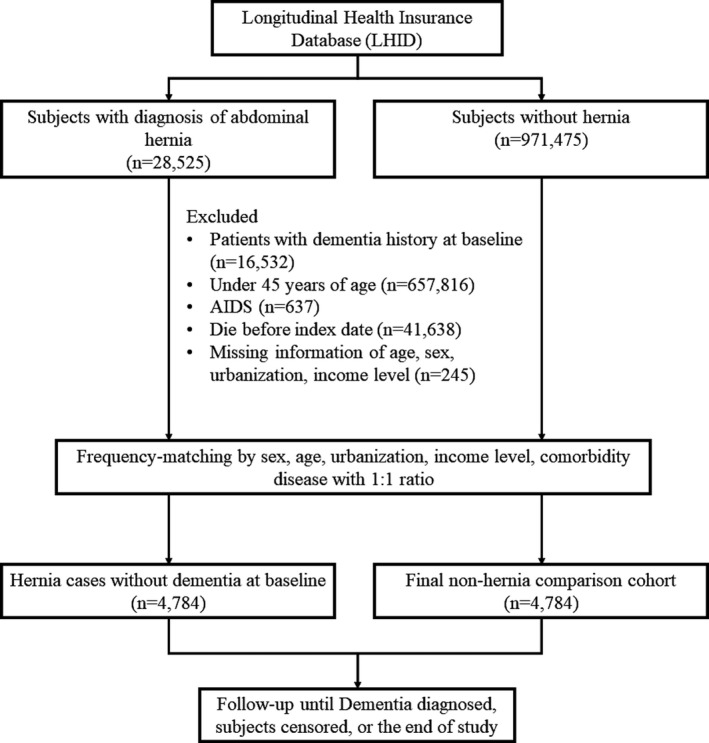
Flow chart for selection of study patients and follow‐up process. AIDS, acquired immunodeficiency syndrome

Patients aged 45 years and older with the diagnosis of abdominal hernias (ICD‐9‐CM codes 550.xx‐553.xx) between January 1, 2000, and December 31, 2008, were included as the hernia group (Figure 1). The database was searched for the study population to identify any hospitalized event with hernia as one of the discharge diagnoses and used outpatient claims to identify any visit for hernia. Patients were classified as having hernia if they had two or more outpatient visits with a hernia diagnostic code or at least one hospital admission with a diagnostic code of hernia within 365 calendar days. The first date of outpatient visit or hospital admission date that met the selection criteria for hernias, whichever came first, was considered the index date. Patients who had a history of dementia (ICD‐9‐CM codes 290, 294.1, and 331.0, respectively) diagnosed before or on the index date, died before index date as well as those with missing information (e.g., age or sex) were excluded. Besides, patients with a history of acquired immunodeficiency syndrome were also excluded. For each patient with a hernia, one insured patient without a history of hernia or diseases (i.e., dementia or acquired immunodeficiency syndrome) before and on the index date were randomly assigned to the nonhernia group. Frequency matching with age, sex, urbanization level, socioeconomic status, and all comorbidities at ratio of 1:1 was adopted for the case and comparison cohort.

The baseline comorbidities of hypertension (ICD‐9‐CM 401‐405), diabetes mellitus (DM, ICD‐9‐CM 250), chronic obstructive pulmonary disease (COPD, ICD‐9‐CM 491, 492, and 496), depression (ICD‐9‐CM 296.2, 296.3, 296.82, 300.4, and 311), coronary artery disease (CAD, ICD‐9‐CM 410‐414), head injury (ICD‐9‐CM 310.2, 800, 801, 803, 804, 850, 851, 853, and 854), stroke (ICD‐9‐CM 430‐438), chronic kidney disease (CKD, ICD‐9‐CM 580, 581–589, 753, 403, 404, 250.4, 274.1, 440.1, 442.1, 447.3, 572.4, 642.1, and 646.2), congestive heart failure (CHF, ICD‐9‐CM 428), obesity (ICD‐9‐CM 278.00, 278.01 278, 649.1, and 783.1), hyperlipidemia (ICD‐9‐CM 272), alcoholism‐related disease (ICD‐9‐CM 291, 303, 305, 571.0, 571.1, 571.2, 571.3, 790.3, and V11.3), and smoking (ICD‐9‐CM 305.1, 649.0, and 490–496) were identified. In addition, Charlson Comorbidity Index (CCI; Charlson, Pompei, Ales, & MacKenzie, 1987), which contains 17 weighted comorbidities, was also calculated for each participant. Definition of comorbidities was the presence of diagnosis codes in at least two outpatient claims or one inpatient claim. For the socioeconomic status, we classified those with a well‐defined monthly wage into three categories: New Taiwan dollar (NTD) <20,000, 20,000–39,999, ≥40,000. The urbanization level was categorized into four levels according to population density of the residential area defined by the Taiwanese National Institutes of Health with level 1 being the most urbanized and level 4 being the least urbanized (Liu et al., 2006).

### 2.3 Study outcomes and follow‐up

Our primary outcomes were pooled incidence rates of dementia defined as at least two outpatient visits or one hospital admission with related diagnosis codes, in patients with or without hernias. All participants were followed till the occurrence of dementia, termination of study follow‐up (i.e., December 31, 2013), or death (i.e., withdrawal from the insurance system), whichever came first.

### 2.4 Statistics

Because this was a retrospective study, we did not a priori power calculations. Baseline characteristics were presented as mean (standard deviation) or numbers (percent), and group difference was measured by *t* test or Pearson *χ* ^2^ test for continuous and categorical variables, respectively. The incidence rate of dementia was defined as the total number of dementia diagnosis divided by the total sum of follow‐up years (i.e., per 1,000 person‐years), and the cumulative incidence curves were compared by Kaplan–Meier method with the difference being tested by log‐rank test between hernia and comparison cohort. Cox proportional hazard model was applied in the study to estimate the hazard ratios (HRs) and 95% confidence intervals (CIs) of primary outcomes. For multivariate models, the study adjusted age, sex, and any significant variables in crude model for the outcome. All statistical analyses were performed with SAS 9.4 software (SAS Institute Inc.) and R software (R Foundation for Statistical Computing). Statistical significance was defined as a two‐tailed *p*‐value <.05.

## 3 RESULTS

**Figure 2:**
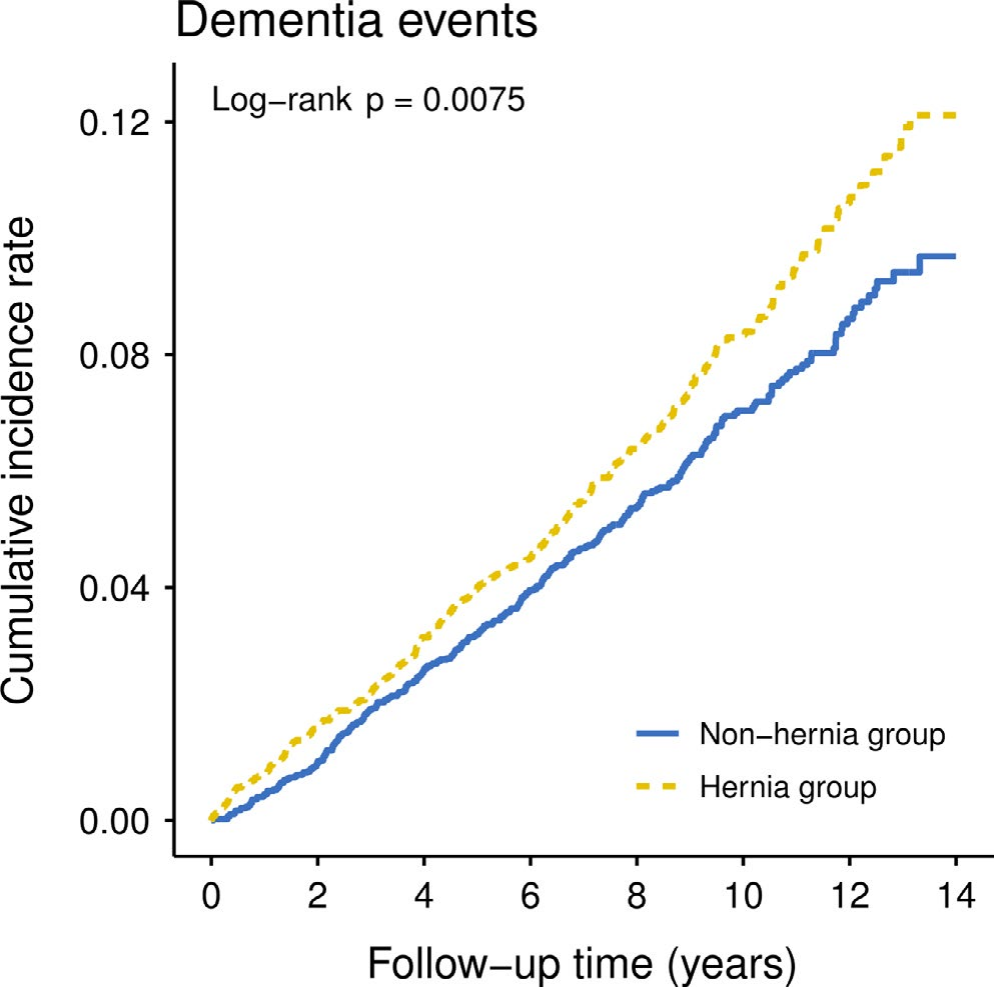
Kaplan–Meier model‐estimated cumulative incidence of dementia in hernia and nonhernia groups in the follow‐up years

The study included 4,784 newly diagnosed hernia participants, and 4,784 nonhernia participants after matching, and the demographic characteristics were shown in Table 1. There were no differences in distribution of age group, sex, socioeconomic status, urbanization level, and all comorbidities between two cohorts. There was higher prevalence in age group (46–64 years, 54.93% vs. 54.72%), male (both 82.44%), socioeconomic status (NTD < 20,000, both 57.4%), urbanization level (level 2, both 30.2%) in hernia, and comparison cohort. The mean follow‐up intervals in the hernia participants and nonhernia participants were 8.81 ± 3.22 and 9.01 ± 3.06 years (*p* = .0024), respectively. With a reference group of nonhernia participants, significant higher risk of dementia was observed in hernia participants (HR, 1.23; 95% CI, 1.06–1.43; *p* = .0077) in crude Cox proportional hazard model; moreover, the hernia participants showed consistent result with crude model in the multivariate model after adjusting age group, sex, and significant variables in crude model, namely socioeconomic status, hypertension, diabetes, coronary artery disease, stroke, COPD, CKD, CHF, hyperlipidemia, smoking, and CCI (Table 2). In addition, advanced age, hypertension, head injury, and stroke were found to be significant risk factors for dementia, while a high socioeconomic status and history of coronary artery disease appeared to be protective against the development of the disorder in our cohort study (Table 2). Table 3 presented the subgroup analysis of dementia risk between hernia and nonhernia cohort. Compared with nonhernia cohort, participants who diagnosed with hernia had significant higher risk to develop dementia in male gender, socioeconomic status of <20,000, urbanization level 1, with and without congestive heart failure, without hypertension, with comorbidities of diabetes, depression, CAD, head injury, stroke, COPD, CKD, hyperlipidemia, obesity, smoking, and alcoholism‐related disease. Figure showed the Kaplan–Meier curve for the cumulative risk of dementia for hernia and nonhernia cohort, where a significant difference was observed between the two cohorts (*p* = .0075 by log‐rank test, Figure 2).

**Table 1:** Comparison of demographics and comorbidity between hernia patients and controls

	Hernia				*p*‐Value^b^
	No *n* = 4,784		Yes *n* = 4,784		
	*n*	%	*n*	%	
Age, years					
45–64	2,628	54.93	2,618	54.72	.8649
65–74	1,307	27.32	1,297	27.11	
≥75	849	17.75	869	18.16	
Mean (*SD*)	63.18 (11.13)		63.46 (11.05)		.2201^a^
Gender					
Female	840	17.56	840	17.56	1.0000
Male	3,944	82.44	3,944	82.44	
Socioeconomic status (NTD)					
<20,000	2,746	57.40	2,746	57.40	1.0000
20,000–39,999	1,404	29.35	1,404	29.35	
≥40,000	634	13.25	634	13.25	
Urbanization level^c^					
Level 1	1,390	29.06	1,390	29.06	1.0000
Level 2	1,445	30.20	1,445	30.20	
Level 3	729	15.24	729	15.24	
Level 4	1,220	25.50	1,220	25.50	
Comorbidity					
Hypertension	1,903	39.78	1,903	39.78	1.0000
Diabetes	586	12.25	586	12.25	1.0000
Coronary artery disease	1,061	22.18	1,061	22.18	1.0000
Head injury	85	1.78	85	1.78	1.0000
Depression	51	1.07	51	1.07	1.0000
Stroke	410	8.57	410	8.57	1.0000
Chronic obstructive pulmonary disease	1,001	20.92	1,001	20.92	1.0000
Chronic kidney disease	303	6.33	303	6.33	1.0000
Congestive heart failure	126	2.63	126	2.63	1.0000
Hyperlipidemia	722	15.09	722	15.09	1.0000
Obesity	2	0.04	2	0.04	1.0000
Smoking	1,300	27.17	1,300	27.17	1.0000
Alcoholism‐related disease	22	0.46	22	0.46	1.0000
Charlson Comorbidity Index					
0	1,332	27.84	1,332	27.84	1.0000
1	834	17.43	834	17.43	
≥2	2,618	54.72	2,618	54.72	

Note

Data presented as mean ± *SD*, or *n* (%).

Abbreviations: NTD, new Taiwan dollar; *SD*, standard deviation.

a *t* test.

b Chi‐square test.

c Urbanization levels categorized into 4 levels by population density of the residential area with level 1 being the most urbanized and level 4 being the least urbanized.

**Table 2:** Cox model with hazard ratios and 95% confidence intervals of dementia‐associated hernia and other factors in study population

Variable	Dementia no. (*n* = 682)	Crude^a^			Adjusted^b^		
		HR	95% CI	*p*‐Value	HR	95% CI	*p*‐Value
Hernia							
No	310	1.00	Reference		1.00	Reference	
Yes	372	1.23	1.06, 1.43	.0077	1.24	1.07, 1.45	.0047
Gender							
Female	125	1.00	Reference		1.00	Reference	
Male	557	1.00	0.82, 1.22	.992	0.91	0.75, 1.11	.3667
Age, years							
45–64	101	1.00	Reference		1.00	Reference	
65–74	283	5.90	4.70, 7.41	<.0001	4.80	3.79, 6.09	<.0001
≥75	298	11.04	8.81, 13.83	<.0001	8.00	6.23, 10.28	<.0001
Socioeconomic status							
<20,000	523	1.00	Reference		1.00	Reference	
20,000–39,999	143	0.53	0.44, 0.63	<.0001	0.78	0.64, 0.94	.0089
≥40,000	16	0.13	0.08, 0.22	<.0001	0.41	0.24, 0.68	.0006
Urbanization							
Level 1	198	1.00	Reference				
Level 2	194	0.91	0.75, 1.11	0.3558			
Level 3	94	0.87	0.68, 1.11	0.27			
Level 4	196	1.07	0.88,1.31	0.4785			
Comorbidities (Yes vs. No)							
Hypertension	393	2.37	2.04, 2.76	<.0001	1.27	1.05, 1.53	.0139
Diabetes	114	1.62	1.32, 1.98	<.0001	1.18	0.94, 1.48	.1605
Coronary artery disease	226	1.90	1.62, 2.23	<.0001	0.81	0.67, 0.98	.0292
Head injury	23	2.18	1.44, 3.30	.0002	2.17	1.42, 3.30	.0003
Depression	7	1.14	0.54, 2.41	.7264			
Stroke	122	2.81	2.31, 3.42	<.0001	1.25	1.01, 1.56	.041
COPD	250	2.55	2.18, 2.98	<.0001	1.23	0.87, 1.75	.247
Chronic kidney disease	65	1.83	1.42, 2.36	<.0001	1.15	0.88, 1.51	.3019
Congestive heart failure	28	1.90	1.30, 2.77	.0009	0.75	0.50, 1.11	.1522
Hyperlipidemia	118	1.44	1.18, 1.76	.0004	1.19	0.95, 1.49	.1279
Obesity	0						
Smoking	287	2.23	1.91, 2.60	<.0001	1.11	0.79, 1.56	.5559
Alcoholism‐related disease	0						
Charlson Comorbidity Index							
0	129	1.00	Reference		1.00	Reference	
1	105	1.41	1.09, 1.83	.0088	1.08	0.83, 1.41	.5757
≥2	448	2.18	1.79, 2.65	<.0001	0.98	0.76, 1.25	.8462

Abbreviation: COPD, chronic obstructive pulmonary disease.

a Crude HR represented relative hazard ratio.

b Adjusted hazard ratio: Multivariate Cox regression model adjusted for age, gender, and significant variables in crude Cox regression model.

**Table 3:** Incidence and Cox proportional hazard regression with hazard ratios and 95% confidence intervals of dementia associated with and without hernia by gender, age group, and comorbidities

Variable	Hernia						Compared to Control	
	No			Yes			Crude HR	Adjusted HR^a^
	Event	PY	IR	Event	PY	IR		
Total	310	43,095	7.19	372	42,164	8.82	1.23 (1.06, 1.43)**	1.24 (1.07, 1.45)**
Gender								
Female	57	7,913	7.20	68	7,652	8.89	1.24 (0.87, 1.76)	1.27 (0.89, 1.80)
Male	253	35,182	7.19	304	34,512	8.81	1.23 (1.04, 1.45)*	1.24 (1.05, 1.47)*
Age group, year								
45–64	47	24,552	1.91	54	24,230	2.23	1.16 (0.79, 1.72)	1.15 (0.78, 1.70)
65–74	128	11,815	10.83	155	11,388	13.61	1.27 (1.00, 1.60)*	1.26 (1.00, 1.59)
≥75	135	6,728	20.06	163	6,545	24.9	1.24 (0.99, 1.56)	1.24 (0.98, 1.56)
Socioeconomic status								
<20,000	236	24,744	9.54	287	23,920	12.00	1.26 (1.06, 1.49)**	1.28 (1.07, 1.52)**
20,000–39,999	69	12,705	5.43	74	12,696	5.83	1.07 (0.77, 1.49)	1.03 (0.74, 1.43)
≥40,000	5	5,647	0.89	11	5,548	1.98	2.25 (0.78, 6.49)	2.48 (0.85, 7.22)
Urbanization								
Level 1	84	12,237	6.86	114	11,908	9.57	1.40 (1.05, 1.85)*	1.45 (1.09, 1.93)**
Level 2	88	13,111	6.71	106	12,775	8.30	1.24 (0.93, 1.64)	1.26 (0.95, 1.68)
Level 3	43	6,595	6.52	51	6,513	7.83	1.20 (0.80, 1.80)	1.24 (0.82, 1.86)
Level 4	95	11,152	8.52	101	10,969	9.21	1.08 (0.82, 1.43)	1.07 (0.81, 1.42)
Comorbidities								
Hypertension								
No	129	27,128	4.76	160	26,690	5.99	1.26 (1.00, 1.59)*	1.25 (0.99, 1.57)
Yes	181	15,967	11.34	212	15,473	13.7	1.21 (0.99, 1.47)	1.24 (1.02, 1.51)*
Diabetes								
No	261	38,295	6.82	307	37,436	8.20	1.20 (1.02, 1.42)*	1.22 (1.03, 1.44)*
Yes	49	4,801	10.21	65	4,728	13.75	1.34 (0.93, 1.94)	1.37 (0.94, 1.99)
CAD								
No	201	34,099	5.89	255	33,431	7.63	1.3 (1.08, 1.56)**	1.30 (1.08, 1.56)**
Yes	109	8,996	12.12	117	8,733	13.40	1.11 (0.85, 1.44)	1.13 (0.87, 1.46)
Head injury								
No	298	42,416	7.03	361	41,476	8.70	1.24 (1.06, 1.45)*	1.26 (1.08, 1.47)**
Yes	12	679	17.68	11	688	16.00	0.90 (0.4, 2.05)	0.81 (0.34, 1.95)
Depression								
No	306	42,698	7.17	369	41,772	8.83	1.23 (1.06, 1.44)**	1.25 (1.08, 1.46)**
Yes	4	397	10.07	3	392	7.66	0.78 (0.17, 3.49)	0.29 (0.04, 2.35)
Stroke								
No	253	39,962	6.33	307	39,058	7.86	1.24 (1.05, 1.47)*	1.26 (1.06, 1.48)**
Yes	57	3,133	18.19	65	3,106	20.93	1.15 (0.80, 1.63)	1.14 (0.80, 1.63)
COPD								
No	192	34,910	5.50	240	34,317	6.99	1.27 (1.05, 1.54)*	1.28 (1.06, 1.55)*
Yes	118	8,185	14.42	132	7,847	16.82	1.17 (0.91, 1.50)	1.18 (0.92, 1.52)
CKD								
No	281	40,707	6.90	336	39,818	8.44	1.22 (1.04, 1.43)*	1.24 (1.06, 1.45)**
Yes	29	2,388	12.14	36	2,346	15.34	1.26 (0.77, 2.05)	1.28 (0.78, 2.08)
CHF								
No	302	42,083	7.18	352	41,270	8.53	1.19 (1.02, 1.39)*	1.20 (1.03, 1.40)*
Yes	8	1,012	7.90	20	894	22.36	2.79 (1.23, 6.35)*	2.97 (1.29, 6.83)*
Hyperlipidemia								
No	251	37,482	6.7	313	36,615	8.55	1.28 (1.08, 1.51)**	1.29 (1.10, 1.53)**
Yes	59	5,614	10.51	59	5,549	10.63	1.01 (0.70, 1.45)	1.05 (0.73, 1.50)
Obesity								
No	310	43,080	7.20	372	42,148	8.83	1.23 (1.06, 1.43)**	1.24 (1.07, 1.45)**
Yes	0							
Smoking								
No	175	32,265	5.42	220	31,737	6.93	1.28 (1.05, 1.56)*	1.29 (1.05, 1.57)*
Yes	135	10,830	12.47	152	10,427	14.58	1.17 (0.93, 1.48)	1.20 (0.95, 1.51)
ARD								
No	310	42,941	7.22	372	42,022	8.85	1.23 (1.06, 1.43)**	1.24 (1.07, 1.45)**
Yes	0							
CCI								
0	54	13,271	4.07	75	13,121	5.72	1.40 (0.99, 1.99)	1.36 (0.96, 1.93)
1	48	7,804	6.15	57	7,624	7.48	1.23 (0.83, 1.80)	1.26 (0.86, 1.86)
≥2	208	22,020	9.45	240	21,419	11.20	1.19 (0.99, 1.43)	1.20 (1,0.00 1.45)

Abbreviations: ARD, alcoholism‐related disease; CAD, coronary artery disease; CCI, Charlson Comorbidity Index; CHF, congestive heart failure; CI, confidence interval; CKD, chronic kidney disease; COPD, chronic obstructive pulmonary disease; HR, hazard ratio; IR, incidence rates, per 1,000 person‐years; PY, person‐years.

a Adjusted hazard ratio: mutually adjusted for gender, age, and baseline comorbidities in Cox proportional hazard regression.

* *p*‐Value < .05.

** *p*‐Value < .01.

## 4 DISCUSSION

With a rapid increase in the aging populations worldwide, dementia has become a growing public health problem for which early detection may offer a therapeutic window. Identification of etiological factors for dementia with timely implementation of active interventions targeting the modifiable factors in high‐risk populations may postpone or even prevent clinical onset of the disorder. To date, no published clinical evidence has previously demonstrated a correlation between hernia and dementia. Our study identified hernia as an independent variable associated with a 1.24‐fold increase in risk for dementia development (*p* = .0047), even after adjustment for sex, age, socioeconomic status, urbanization, and medical comorbidities. Furthermore, advanced age, hypertension, head injury, and stroke were shown to be significant risk factors for dementia, while a high socioeconomic status and history of coronary artery disease appeared to be protective against development of the disorder in our cohort study. Although the finding of current study (i.e., 1.24‐fold increase in risk for dementia) may not allow clinicians to select subjects for dementia screening programs, our results highlighted a possible link between hernia and the development of dementia.

In our patients without hernia, the incidence of dementia was 7.19 cases per 1,000 person‐years, which is comparable with that in a previous study (i.e., 9.87 cases per 1,000 person‐years; Chan et al., 2013). The age‐adjusted prevalence of all‐cause dementia has been reported to be 8.04% in Taiwan (Sun et al., 2014). It was estimated that the dementia‐stricken population was up to 36.5 million worldwide in 2010, with 7.7 million new cases being diagnosed each year (i.e., a new case every 4 s; Sosa‐Ortiz, Acosta‐Castillo, & Prince, 2012). In the United States, official death records in 2013 showed 84,767 deaths from AD, making it the sixth leading killer and the fifth leading cause of death among the elderly (i.e., ≥ 65 years; Alzheimer's Association, 2016). Productivity equivalent to more than $221 billion was wasted in 2015 alone as over 15 million family members as well as other unpaid caregivers devoted themselves to the care of American populations afflicted with Alzheimer and other dementias (Alzheimer's Association, 2016).

Despite current advances in the diagnosis of mental diseases, diagnosis of dementia remains challenging to physicians in primary care and is prone to be delayed or missed (Boustani et al., 2005). There is no previous study reporting the possible relationship between hernia and dementia. Taking into account the action of MMP, which is a possible link between the two clinical entities, the present study attempted to identify their possible association. The results showed a 1.24‐fold increase in risk for subsequent development of dementia in patients with hernias compared to those without. These findings carried several striking clinical implications. First, in view of the potential risk for subsequent dementia, potential screening strategies may be implemented for early detection of the disease for high‐risk patients (e.g., advanced age) with co‐existing hernia. Second, patients with hypertension, head injury, or stroke may also be chosen for dementia screening programs. Consistent with the findings of the present study, satisfactory cardiovascular risk factor control has been reported to delay or even prevent the development of dementia (Patterson et al., 2008). Third, MMP may be a potential target in the treatment of dementia, although the clinical significance remains to be elucidated.

Multivariate analysis showed that patients with advanced age (≥65 years), low socioeconomic status, hypertension, head injury, and stroke exhibited significantly higher risks of developing dementia, after adjusting for covariates. The findings support those from previous studies identifying these comorbidities as potential risk factors for dementia (Goldbourt, Schnaider‐Beeri, & Davidson, 2007; Jorm & Jolley, 1998; Kohler et al., 2015; Poblador‐Plou et al., 2014; Qiu et al., 2010; Sahathevan et al., 2012). However, the finding of coronary artery disease being protective against the development of dementia in the current study remains poorly explained. In addition, there was no significant gender difference in dementiariskin the present cohort (Table 2). While the finding was in concert with that of some previous studies (Jorm & Jolley, 1998; Yang et al., 2016), it was different from that of other studies reporting a higher risk of Alzheimer disease or dementia in women than in men (Gao, Hendrie, Hall, & Hui, 1998; Sun et al., 2014).

Hernia formation and recurrence are often linked to distorted collagen metabolism caused by MMPs in fascia transversalis (Antoniou et al., 2011, 2009; Franz, 2008; Henriksen et al., 2011). In the brain, MMPs play multiple roles ranging from synaptic plasticity, brain development, maintaining normal physiological functions, and recovery after injury (Kim & Joh, 2012; Mroczko et al., 2013; Rempe et al., 2016; Rosenberg, 2009; Weekman & Wilcock, 2016). On the other hand, accumulating evidence suggests that MMPs may play a pivotal role in the pathogenesis of several neurodegenerative disorders including multiple sclerosis, AD, Parkinson's disease, malignant glioma, neuroinflammation, and ischemia (Kim & Joh, 2012; Mroczko et al., 2013; Rempe et al., 2016; Rosenberg, 2009; Weekman & Wilcock, 2016). Although the mechanism remains unclear, dysregulation of MMPs may be an important factor linking hernia and dementia.

The major strength of the current investigation was the utilization of a nationwide population‐based database as well as the longitudinal, observational design together with a lengthy follow‐up. For the purpose of the present study, all insurance claims were examined and coded by trained medical reimbursement staff as well as peer‐reviewed in accordance with the standard criteria for diagnoses. Because incorrect diagnoses or erroneous coding would result in heavy penalties for physicians, the diagnoses and coding used in this study should be highly reliable. In addition, selection biases were minimized because NHIRD are highly representative of the health information of Taiwan's general population. Risks had been well adjusted in the present study. Besides, statistical power was reinforced by the large sample size to consolidate the results. Furthermore, the longitudinal design allowed elucidation of the temporal association between dementia and hernia, which could help establish a potential positive correlation.

On the other hand, subgroup analysis was not performed on different types of abdominal hernias because inguinal hernia is known to constitute the large majority of cases. Although MMPs may have a role to play in the recurrence of inguinal hernia, (Antoniou et al., 2011, 2009) we did not analyze the association between dementia and inguinal hernia recurrence because several modifiable and nonmodifiable contributors to hernia recurrence including perioperative (e.g., the surgeon—learning curve and caseload), patient (age, body mass index), and hernia (e.g., direct inguinal hernias vs. indirect inguinal hernias) factors have been reported (Ashrafi, Siddaiah‐Subramanya, Memon, & Memon, 2019). Besides, taking into account the limited sample size of our study, an estimated rate of 13% for surgical repair of recurrent inguinal hernias among all inguinal hernia operations (Ashrafi et al., 2019) would further diminish the study population and blemish the accuracy of risk assessment. Additionally, despite being a common clinical observation, the association between increase in abdominal pressure (e.g., chronic cough and constipation) and the development of hernias remains controversial (Ruhl, 2007). Although previous studies reported the presence of obstipation (Liem, van der Graaf, Zwart, Geurts, & van Vroonhoven, 1997) or chronic cough (Ruhl, 2007) may increase the risk of inguinal hernia in female patients, this association was not found in their male counterparts in a large‐scale study (Ruhl, 2007). Also, taking into consideration possible bias arising from listing the diagnoses of “chronic cough” and “chronic constipation” as independent variables for analysis because of the lack of clear definitions, these conditions were not included for further evaluation.

Caution should be taken in the interpretation of our results due to several limitations in the current study. First, our study has the inherent limitations pertaining to the use of a retrospective database (e.g., selection bias and misclassification bias). Second, as the symptom of dementia may be unrecognized and the demented patients may not seek active medical care, some cases with dementia may be missed. However, it could be assumed that the probability of missing the diagnosis was the same for both groups in our study because of the large sample size. Third, even though socioeconomic factors and urbanization level of the enrollees have been considered in this study, levels of education (Karp et al., 2004; Sun et al., 2014) and social disengagement (Bassuk, Glass, & Berkman, 1999), which are known confounders for dementia, were not available in the NHIRD. In addition, previous occupation, physical activity, family history of hernia, and mobility are also potential confounders for hernia. Matching for these confounders would have improved the validity of our results. Fourth, information on laboratory studies, imaging findings, or symptom severity of dementia, which were inherent limitations of the insurance claims data, was unavailable for further analysis. Fifth, matching comorbidities for subject selection also meant reduction of sample size and increased risk for bias. Additionally, the incidence of dementia may vary in HIV‐infected patients depending on whether antiretroviral therapy is used (Larussa et al., 2006). To avoid biasing our results, HIV‐infected patients were excluded.

In conclusion, the results of the current nationwide, population‐based longitudinal study provided evidence in support of a temporal association between abdominal wall hernia and the risk of dementia irrespective of the subjects' age, sex, socioeconomic factors, urbanization, and comorbidities. Advanced age (i.e., ≥65 years), low socioeconomic status (<20,000), hypertension, head injury, and stroke showed significant associations with dementia. Despite these findings, the results of our study should be validated in further prospective studies with larger samples.

## CONFLICT OF INTEREST

All authors report no conflicts of interest.

## AUTHOR CONTRIBUTIONS

The authors' contributions are as follows. Kuo‐Chuan Hung and Chia‐Hung Kao involved in the conception and design of the study. Chia‐Hung Kao performed the administrative support. All authors collected the data, organized, performed the data analysis and interpretation, wrote the manuscript, and finally approved the manuscript.

## Data Availability

The dataset used in this study is held by the Taiwan Ministry of Health and Welfare (MOHW). The Ministry of Health and Welfare must approve our application to access these data. Any researcher interested in accessing this dataset can submit an application form to the Ministry of Health and Welfare requesting access. Please contact the staff of MOHW (Email: stcarolwu@mohw.gov.tw) for further assistance. Taiwan Ministry of Health and Welfare Address: No. 488, Sec. 6, Zhongxiao E. Rd., Nangang Dist., Taipei City 115, Taiwan (R.O.C.). Phone: +886‐2‐8590‐6848. All relevant data are within the paper.
